# Case report: Aorto-left atrial fistula—A rare complication of native aortic valve endocarditis

**DOI:** 10.1186/s43044-023-00384-z

**Published:** 2023-07-11

**Authors:** Sheema Saadia, Fateh Ali Tipoo Sultan, Sara Iqbal, Saulat Hasnain Fatimi, Aiysha Nasir

**Affiliations:** 1 Section of Cardiology, Department of Medicine, The Aga Khan University Hospital, Karachi, Pakistan; 2 Section of Cardiothoracic Surgery, Department of Surgery, The Aga Khan University Hospital, Karachi, Pakistan

**Keywords:** Aortic endocarditis, Valvular heart disease, Transesophageal echocardiography, Surgical treatment, Dual valve replacement (DVR)

## Abstract

**Background:**

Aorto-cavitary fistula is a rare complication of infective endocarditis. Multimodal imaging is commonly required to assess the severity and extent of infection because of the complex pathology of the valvular and paravalvular apparatus in endocarditis.

**Case presentation:**

We present an unusual case of a middle-aged man with recent history of meningoencephalitis who developed infective endocarditis complicated by ruptured abscess in inter-valvular fibrosa between aortic and mitral valve resulting in free communication or fistula formation between aorta and left atrium. Patient underwent double valve replacement (aortic and mitral) along with repair of the aorta.

**Conclusions:**

Our case highlights recognition of this rare clinical presentation of aorto-left atrial fistula in infective endocarditis and the diagnostic role of transesophageal echocardiography in good clinical outcome with aggressive and timely management.

## Background

Despite advancements in diagnostic modalities, antimicrobial and surgical treatment, infective endocarditis (IE) still heralds a grave prognosis particularly due to complications resulting rapid deterioration. Prompt recognition, early diagnosis and treatment is paramount in reducing the associated morbidity and mortality. Globally, incidence of IE had been reported to be approximately 1.7–6.2 cases per 100,000 per year and even up to 32.4 cases per million in some parts of the world [1, 2]. Overall, in-hospital mortality rate is 17–22.7% [2, 3]. In Pakistan, annual number of infective endocarditis episodes is 0.99 per 1000 hospital admissions and in-hospital mortality was reported 27% in a single center study in Pakistan [4]. Since over 50% of patients require operative management which can significantly affect the quality of life, the associated morbidity rates are also high [5, 6].

IE can cause wide spectrum of complications including stroke, embolization, heart failure, conduction abnormality, abscess, pseudoaneurysm formation and the very rare fistula formation as a result of spread of infection from valvular to peri-valvular tissues. This abscess can progress into pseudoaneurysm formation that is why both are difficult to differentiate clinically and can present as asymptomatic (9%) to a wide range of associated complications like symptoms and/or signs of active endocarditis (39%), followed by dyspnea and heart failure (16%), chest pain (10%), cerebrovascular accident and systemic embolism (12%) [7]. Transesophageal echocardiography is a better modality that differentiates abscess from pseudoaneurysm, i.e., pseudoaneurysm appears as a pulsatile echo-free pouch located between posterior aortic root and anterior mitral leaflet, expanding during systole and collapsing during diastole with an aliased Doppler flow of brief duration from LVOT into pseudoaneurysm. In comparison with pseudoaneurysm, abscess is located at anterior or posterior aortic ring, smaller in size, does not exhibit pulsatility and color Doppler flow imaging shows no flow through the abscess [8]. The fistula formation is particularly observed in aortic valve involvement since internal rupture of abscesses and pseudo-aneurysms can result in formation of aorto-cavitary fistulae (ACF). These aorto-cavitary fistulae can create intracardiac shunts causing rapid clinical deterioration and hemodynamic instability. However, this complication in infective endocarditis is rare and has been reported in estimated 1–2% of all cases [9]. Here, we report an unusual case of infective endocarditis complicated by abscess in intervalvular fibrosa between aortic and mitral valve followed by rupture and free communication or fistula formation between aorta and left atrium.

## Case presentation

A 49-year-old gentleman presented to clinic with recurrent episodes of fever of up to 101 °F (38.3 C) for the 5 days. He also reported worsening dyspnea and chest heaviness on minimal exertion for the last five days. There was no history of orthopnea, paroxysmal nocturnal dyspnea or syncope. He had history of hypertension and benign prostatic hyperplasia. He had been recently hospitalized and treated for streptococcal meningo-encephalitis and had been discharged 6 days before this clinic visit. Drug history included amlodipine 5 mg once a day, paracetamol and intravenous antibiotics (ceftriaxone and vancomycin in meningitic doses) which he was taking for the last 9 days. On arrival, patient had blood pressure of 135/70 mmHg, heart rate of 80 beats per minute, respiratory rate of 19 breaths per minute and oxygen saturation of 95% on room air. On examination, pulse was regular and there was no neurological deficit or signs of heart failure. Precordial examination revealed a systolic and diastolic murmur along left sternal border. ECG was done that showed no significant abnormalities. He was therefore hospitalized for further work up.

### Investigations

**Fig. 1:**
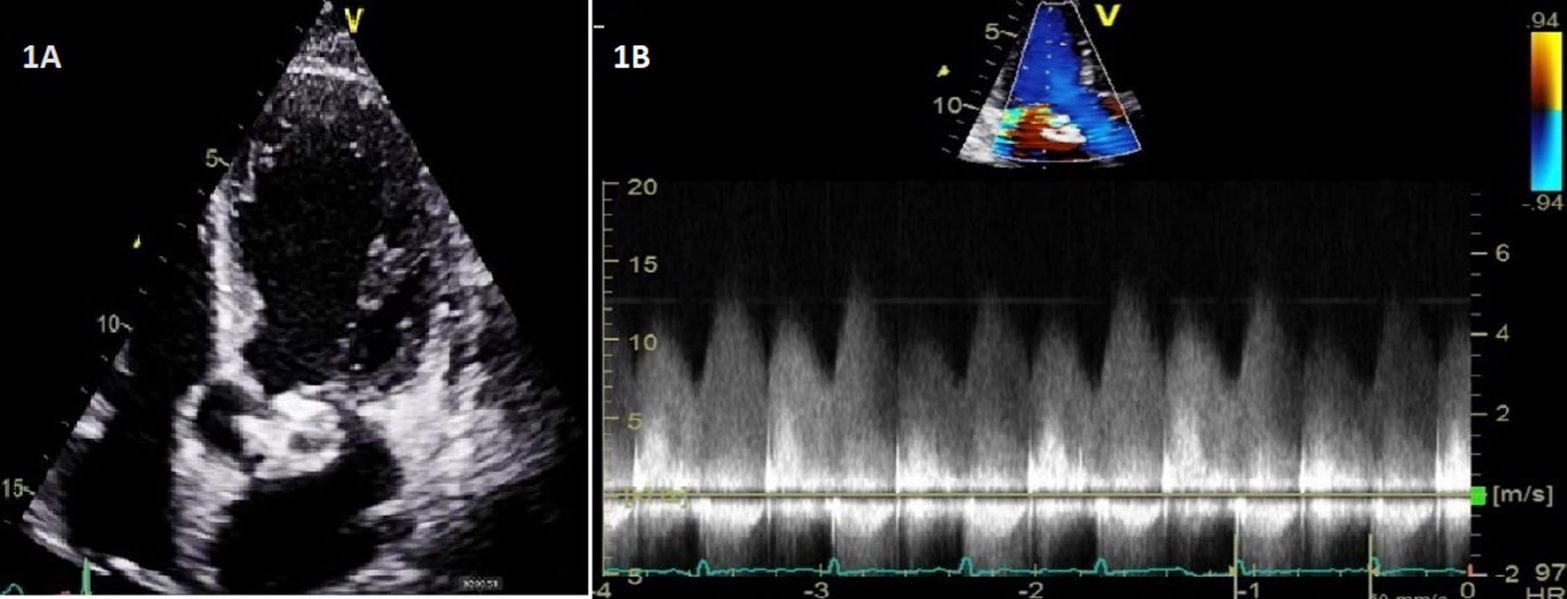
Transthoracic echocardiogram. **A** Apical five-chamber view on transthoracic echo showing vegetations above the aortic valve and an echo-free space in mitral-aortic intervalvular fibrosa consist with abscess. **B** Continuous-wave (CW) Doppler showing continuous flow due to fistula formation between the aorta and left atrium

Laboratory investigation revealed a hemoglobin of 11.6 g/dl, white cell count of 11.7 × 10^9, creatinine of 0.8 mg/dl (cutoff 1.3), Sodium of 134 mmol/L (cutoff 136), Potassium of 4.2 mmol/L, bicarbonate of 21.4 mmol/L (cutoff 21), C-reactive protein of 81 mg/L (0–10), and Nasopharyngeal COVID-19 test was negative. Blood cultures were sent. Transthoracic echocardiogram (TTE) was done which showed normal left ventricular ejection fraction, bicuspid aortic valve with mild aortic stenosis, and small multiple echogenic densities were noted above the aortic valve, suggestive of vegetations. An echo-free space was noted in the mitral-aortic intervalvular fibrosa (MAIVF), consistent with abscess (Fig. 1A). It also showed continuous color flow jet with possibility of fistula formation (Fig. 1B). Transesophageal echo (TEE) was planned, and cardiothoracic surgery team was consulted, but the patient declined further workup and left against medical advice. After one day, he again presented with worsening shortness of breath and was re-hospitalized and planned for surgery after transesophageal echo (TEE). He had heart of 101 beats per minute, blood pressure was 105/65, respiratory rate of 26 breaths per minute and saturation of 84% on room air. He was managed with intravenous furosemide and noninvasive ventilation. So pre-operative TEE was deferred due to his worsening clinical condition. This patient had one positive pathologic criteria, and one major and 3 minor clinical criteria were also positive that supported the diagnosis of a definite infective endocarditis.

### Treatment

**Fig. 2:**
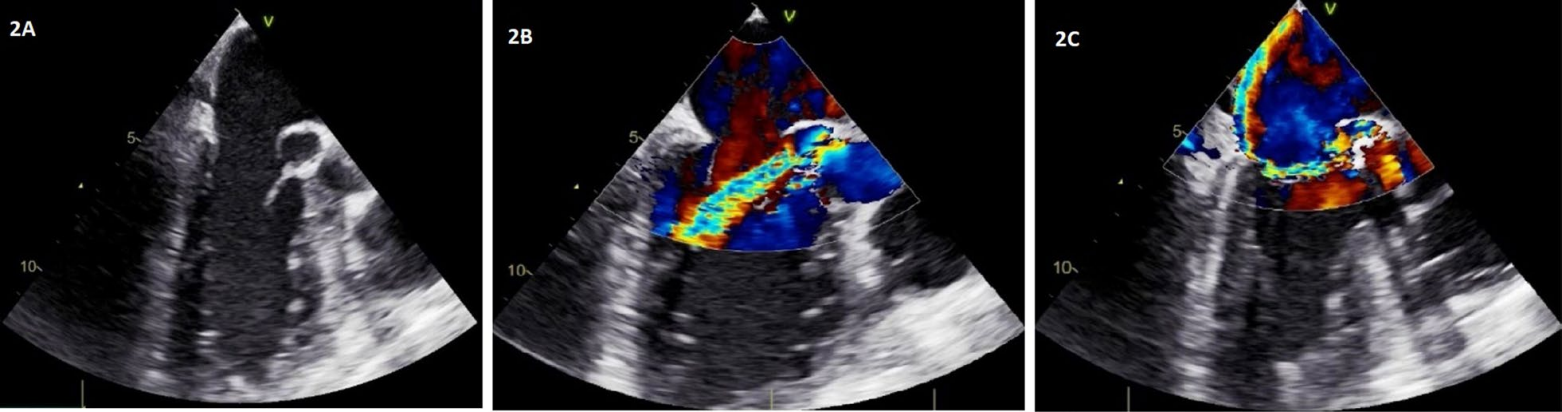
Transesophageal echocardiogram. **A** Transesophageal echo image showing echo-free space with free communication between the aorta and left atrium, consistent with fistula formation. **B** Transesophageal echo image with color Doppler, showing flow through fistulous communication. **C** Transesophageal echo image with color Doppler, showing severe mitral regurgitation with an eccentric posteriorly directed jet. Flow through fistula is also visible

He was taken to the operating room (OR) for the surgery with a plan for intraoperative transesophageal study. TEE was done intraoperatively which confirmed the prior TTE findings of ruptured abscess with fistula formation between aorta and left atrium (Fig. 2A, B). Bicuspid aortic valve with mild aortic stenosis, mild aortic regurgitation and severe, eccentric mitral regurgitation (Fig. 2C). He underwent dual valve replacement (aortic valve 23 mm mechanical & mitral 29 mm mechanical), and aortic repair was also done. Intraoperative findings were abscess between aortic and mitral valve with wide fistula between left coronary sinus and left atrium due to ruptured abscess.

### Outcome and follow-up

**Fig. 3:**
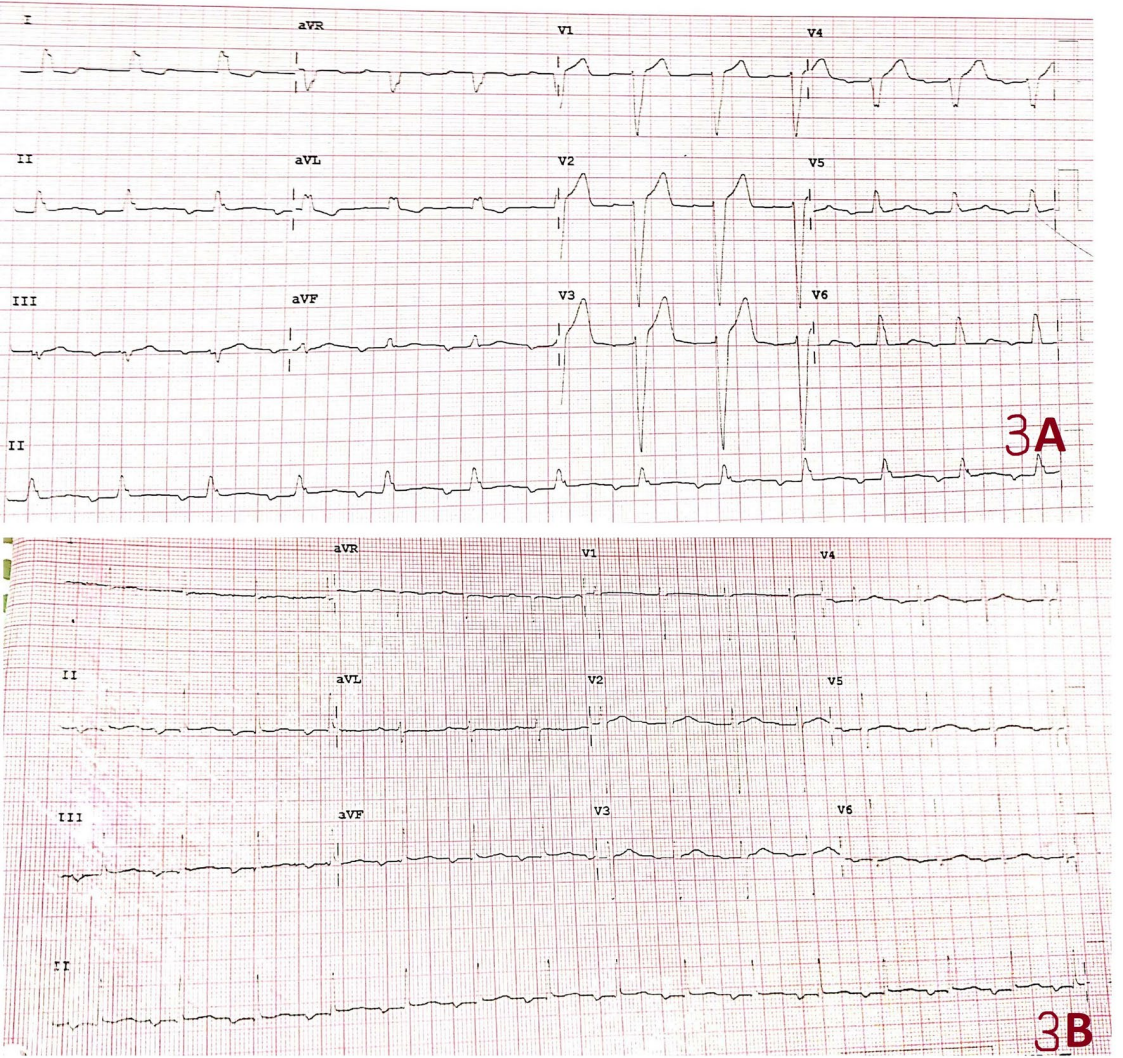
Electrocardiogram (ECG). **A** ECG showing first-degree AV block with underlying left bundle branch block. **B** Subsequent ECG with no left bundle branch block and reduction in PR interval

Post-operatively, he was shifted to cardiac intensive care unit. Ionotropic support (i.e., norepinephrine and vasopressin) was gradually tapered off, and he was successfully extubated on third post-operative day. Intravenous antibiotics (Meropenem and Vancomycin) and anti-coagulation (warfarin) were continued. Post-operatively, his ECG showed first-degree AV block with underlying left bundle branch block (Fig. 3A). Hence, Holter monitoring was done which showed no high degree AV block. Patient was later shifted from surgical intensive care unit to high dependency unit and then, later to ward setup. Subsequent ECG showed no left bundle branch block and reduction in PR interval was also observed as compared to previous ECG (Fig. 3B). His final blood cultures were reported negative. He was eventually discharged after 18 days of hospital stay with complete recovery. On follow-up in clinic after 2 weeks, his symptoms had improved significantly with no recurrence of fever.

#### Microscopic examination was performed that showed

DIAGNOSIS: Aortic wall tissue (container 1): Vessel wall tissue showing marked fibrosis, fibrin and loss of architecture. Negative for granulomatous inflammation and malignancy.

Aortic valve tissue (container 2): Endocardium lined fibromyxoid tissue from the heart valves showing areas of myxoid change, endocardial erosion with lymphoplasmacytic infiltrate, neutrophils, necrosis, fibrinous exudate and hemosiderin laden macrophages compatible with clinical history of infective endocarditis with abscess formation. Negative for granuloma and malignancy.

## Discussion

Mitral-aortic inter-valvular fibrosa (MAIVF), a fibrous structure contiguous to the left ventricular outflow tract (LVOT) between non-coronary cusp of the aortic valve and the anterior mitral leaflet, is necessary for maintaining functional and anatomic integrity [10]. The avascularity and low thickness of this region predisposes it to both injury and infection. Aortic valve endocarditis can lead to the involvement of mitral-aortic inter-valvular fibrosa and the anterior mitral leaflet can occur. These structures can be involved as a consequence of either due to infected aortic regurgitant jet striking their ventricular surfaces or a direct extension and spread of infection from the aortic valve. Moreover, if left untreated, the mitral-aortic intervalvular fibrosa abscess expansion can result in pseudoaneurysm formation and which eventually ruptures into adjacent intracardiac chambers leading to the formation of aorto-cavitary fistulae [9, 11, 12]. Our patient had bicuspid aortic valve which is considered a risk factor for aortic valve IE [13].

In our patient, the route of spread of infection was direct extension as there was only mild AR which was unusual in aortic valve IE as compared to studies done by Anguera and Tribouilloy et al. which showed severe aortic regurgitation (AR) in 50% and 47%, respectively [9, 13]. In comparison with native valve endocarditis, fistula formation is more frequently associated with prosthetic valve endocarditis as Anguera et al. reported a significantly higher occurrence of fistula formation in prosthetic valve endocarditis (5.8 vs. 3.6%, *P* = 0.04). Moreover, these fistula formation case were reported only in aortic valve IE [9]. The fistulae can communicate with the left atrium. In a study done by Anguera et al., 59% of the patients had fistula between left coronary sinus to left atrium which was also consistent with the findings in our patient. In addition, there may be fistula formation between the non-coronary sinus to right ventricle and right coronary sinus to right ventricle [9] which was not observed in our patient.

Ventricular surface involvement of the anterior mitral leaflet through the spread of secondary infection can cause formation of an aneurysm or perforation of anterior mitral leaflet and subsequent mitral regurgitation. However, eccentric mitral regurgitation (MR) can occur without the involvement of anterior mitral leaflet (AML) [11]. Kuroda et al. reported a similar case of IE complicated by the rupture of a pseudoaneurysm of the mitral-aortic intervalvular fibrosa (p-MAIVF) into the left atrium without causing significant AR in a bicuspid aortic valve, but eccentric MR was present without AML involvement [14].

Intracardiac shunting results in volume overload of the left ventricle and subsequently left-sided heart failure and pulmonary edema which was also the case in our patient. The complication rate of aorto-cavitary fistula is high. In a study done by Ignasi et al., heart failure occurrence was higher in patients with aorto-cavitary fistula (52%) as compared to non-ruptured abscess (35%). Moderate-severe heart failure was also found to be an independent predictor of mortality [15]. Heart failure and locally uncontrolled infection warrants urgent (class I indication) surgery in patients with IE which was the case with our patient as well [16].

Early detection, diagnosis, and management of aorto-cavitary fistula requires maintenance of a high index of suspicion for infective endocarditis and its complications. Although transthoracic echocardiography(TTE) is considered the initial diagnostic imaging modality for infective endocarditis; only 50% of fistulous tract can be detected on TTE [13]. However, with transesophageal echocardiography (TEE) the diagnostic yield and rate of detection can be up to 97.8%. Furthermore, in addition to the higher detection rate, due to higher anatomical details previewed and provided in TTE, precise fistula tract characterization in the TEE is possible, and these details are of paramount importance in the pre-operative and intra-operative surgical planning and procedure selection [9, 17]. Aortic paravalvular abscesses may be associated with conduction abnormalities including bundle branch blocks and first-degree, second-degree or high-grade atrioventricular (AV) block which occur in up to 10% of cases [18]. These conduction defects are commonly associated with extension of abscess into the interventricular septum. Our patient developed first-degree AV block which can be explained by perivalvular extension of infection or proximity of aortic valve to Bundle of His which might have been damaged during the surgery.

This case demonstrates a wide spectrum of intracardiac complications such as perivalvular abscess with extension into the MAIVF, aorto-cavitary fistula formation and eccentric MR in infective endocarditis which can be best appreciated with transesophageal echocardiography. It also highlights the possibility of good clinical outcome with early diagnosis and aggressive management.

## Conclusions

Aorto-cavitary fistula formation is a rare and life-threatening complication of infective endocarditis, management of which requires detailed understanding of the complications and imaging findings associated with involvement of MAIVF in IE. In infective endocarditis, timely diagnosis by echocardiography, administration of prompt appropriate antibiotics and early surgical intervention can improve outcomes and reduce complications rate. Earlier detection and prompt management can reduce the mortality and morbidity caused by Aorto-cavitary fistula formation as it is associated with prolonged hospital stay and necessitate surgical exploration and interventions.

## Data Availability

Not applicable.

## Abbreviations

IE: Infective endocarditis
DVR: Dual valve replacement
ACF: Aorto-cavitary fistulae
MAIVF: Mitral-aortic intervalvular fibrosa
TTE: Transthoracic echocardiography
TEE: Transesophageal echocardiography
ECG: Electrocardiogram
LVOT: Left ventricular outflow tract
AR: Aortic regurgitation
AML: Anterior mitral leaflet
MR: Mitral regurgitation
AV: Atrioventricular

## References

[1] Prendergast BD (2006). The changing face of infective endocarditis. Heart.

[2] Selton-Suty C, Célard M, Le Moing V (2012). Preeminence of *Staphylococcus aureus* in infective endocarditis: a 1-year population-based survey. Clin Infect Dis.

[3] Murdoch DR, Corey GR, Hoen B (2009). Clinical presentation, etiology, and outcome of infective endocarditis in the 21st century. Arch Intern Med.

[4] Tariq M, Alam M, Munir G (2004). Infective endocarditis: a five-year experience at a tertiary care hospital in Pakistan. Int J Infect Dis.

[5] Moreillon P, Que Y (2004). Infective endocarditis. Lancet.

[6] Verhagen DWM, Hermanides J, Korevaar JC (2009). Health-related quality of life and posttraumatic stress disorder among survivors of left-sided native valve endocarditis. Clin Infect Dis.

[7] Xie M, Li Y, Cheng TO, Wang X, Lu Q, He L, Fu M (2013). Pseudoaneurysm of the mitral-aortic intervalvular fibrosa. Int J Cardiol.

[8] Ku L, Lv H, Ma X (2022). An abscess of mitral aortic intervalvular fibrosa mimicking an intracardiac mass. J Card Surg.

[9] Anguera I, Miro JM, Vilacosta I (2005). Aorto-cavitary fistulous tract formation in infective endocarditis: clinical and echocardiographic features of 76 cases and risk factors for mortality. Eur Heart J.

[10] Saremi F, Sánchez-Quintana D, Mori S (2017). Fibrous skeleton of the heart: anatomic overview and evaluation of pathologic conditions with CT and MR imaging. Radiographics.

[11] Agrawal A, Amor MM, Iyer D (2015). Aortico-left atrial fistula: a rare complication of bioprosthetic aortic valve endocarditis secondary to *Enterococcus faecalis*. Case Rep Cardiol.

[12] Karalis DG, Bansal RC, Hauck AJ (1992). Transesophageal echocardiographic recognition of subaortic complications in aortic valve endocarditis clinical and surgical implications. Ciculation.

[13] Tribouilloy C, Rusinaru D, Sorel C (2010). Clinical characteristics and outcome of infective endocarditis in adults with bicuspid aortic valves: a multicentre observational study. Heart.

[14] Kuroda M, Yamamoto H, Nakamura Y (2020). Infective endocarditis complicated by pseudoaneurysm of the mitral-aortic intervalvular fibrosa without valvular involvement. JACC Case Reports.

[15] Anguera I, Miro JM, San Roman JA (2006). Periannular complications in infective endocarditis involving prosthetic aortic valves. Am J Cardiol.

[16] Habib G, Lancellotti P, Antunes MJ (2015). ESC Guidelines for the management of infective endocarditis. Eur Heart J.

[17] Hill EE, Herijgers P, Claus P (2007). Abscess in infective endocarditis: the value of transesophageal echocardiography and outcome. Am Heart J.

[18] Choussat R (1999). Perivalvular abscesses associated with endocarditis clinical features and prognostic factors of overall survival in a series of 233 cases. Eur Heart J.

